# Monitoring Hip and Elbow Dysplasia Achieved Modest Genetic Improvement of 74 Dog Breeds over 40 Years in USA

**DOI:** 10.1371/journal.pone.0076390

**Published:** 2013-10-04

**Authors:** Yali Hou, Yachun Wang, Xuemei Lu, Xu Zhang, Qian Zhao, Rory J. Todhunter, Zhiwu Zhang

**Affiliations:** 1 Key Laboratory of Agricultural Animal Genetics and Breeding, National Engineering Laboratory for Animal Breeding, College of Animal Science and Technology, China Agricultural University, Beijing, People’s Republic of China; 2 Laboratory of Disease Genomics and Individualized Medicine, Beijing Institute of Genomics, Chinese Academy of Sciences, Beijing, People’s Republic of China; 3 College of Animal Science and Technology, Northeast Agricultural University, Harbin, People’s Republic of China; 4 Department of Clinical Sciences, College of Veterinary Medicine, Cornell University, Ithaca, New York, United States of America; 5 Institute for Genomic Diversity, Cornell University, Ithaca, New York, United States of America; University of Queensland, Australia

## Abstract

Hip (HD) and Elbow Dysplasia (ED) are two common complex developmental disorders of dogs. In order to decrease their prevalence and severity, the Orthopedic Foundation for Animals (OFA) has a voluntary registry of canine hip and elbow conformation certified by boarded radiologists. However, the voluntarily reports have been severely biased against exposing dogs with problems, especially at beginning period. Fluctuated by additional influential factors such as age, the published raw scores barely showed trends of improvement. In this study, we used multiple-trait mixed model to simultaneously adjust these factors and incorporate pedigree to derive Estimated Breeding Values (EBV). A total of 1,264,422 dogs from 74 breeds were evaluated for EBVs from 760,455 hip scores and 135,409 elbow scores. These EBVs have substantially recovered the reporting bias and the other influences. Clear and steady trends of genetic improvement were observed over the 40 years since 1970. The total genetic improvements were 16.4% and 1.1% of the phenotypic standard deviation for HD and ED, respectively. The incidences of dysplasia were 0.83% and 2.08%, and the heritabilities were estimated as 0.22 and 0.17 for hip and elbow scores, respectively. The genetic correlation between them was 0.12. We conclude that EBV is more effective than reporting raw phenotype. The weak genetic correlation suggested that selection based on hip scores would also slightly improve elbow scores but it is necessary to allocate effort toward improvement of elbow scores alone.

## Introduction

Canine Hip Dysplasia (HD) and Elbow Dysplasia (ED) are inherited developmental joint disorders. The malformation characteristic of both ED and HD leads to osteoarthritis, with the clinical manifestation of lameness or abnormal gait worsening with advancing age. There is an estimated 60–70 million pet dog population in USA households. The prevalence of HD, as estimated by the Orthopedic Foundation for Animals (OFA), varied widely from 1 to 75 percent [1], while the incidence of ED ranged from 1.2 to 47.9 percent in 78 breeds (http://www.offa.org/pdf/elbowarticle.pdf). Because osteoarthritis caused by HD and ED is incurable and progressive, it is increasingly important to improve hip and elbow joint conformation and thus reduce the incidence of osteoarthritis in these joints through selective breeding.

The OFA established a voluntary registry to certify dogs based on their hip and elbow conformations, in order to provide selection criteria and breeding principles for pet dog breeders and owners to lower the occurrence of inherited ED and HD and secondary osteoarthritis in dogs [2]. The radiologists assigned by the OFA score the hip conformation based on the ventrodorsal hip-extended pelvic radiograph recommended by the American Veterinary Medical Association (AVMA Council on Veterinary Service, 1961). The image is scored as excellent, good and fair for so-called “unaffected” dogs, a borderline grade, and grades of mild, moderate and severe for dogs affected with hip dysplasia (www.offa.org/hipgrade). Radiologists also evaluate elbow conformation by employing the protocol established by the International Elbow Working Group, which includes elbow ratings of Normal or Dysplastic Grades I, II or III based on the severity of secondary osteoarthritis presented on an extreme flexed mediolateral projection of the elbow (International Elbow Working Group, 2001) [2]. By the end of 2010, there were 1,066,596 records from 329 breeds reported in the public (voluntary part) OFA database.

Our previous analysis of the combined phenotypes and pedigree in the open access OFA data base showed modestly genetic improvement in hip conformation in Labrador Retriever dogs [3]. The complex inheritance patterns of both ED and HD have been extensively investigated [1], [3]–[7], which supports the argument that in order to reduce the incidence of HD and ED, selection for breeding should be based on estimated breeding values (EBVs) and not simply the phenotype of antecedent generations [3]. The genotype of a dog with a complex trait cannot be revealed from its phenotype. This is particularly relevant for dogs with borderline or unaffected hips that can harbor deleterious mutations that are not apparent from their phenotypes. In our previous study, we have predicted the individual hip score breeding values only in the Labrador Retriever with voluntary OFA records between 1970 to 2007, verifying steady, though modest, genetic improvement over this period, and we provided hip score EBVs in a web-based format for these Labrador retriever owners, breeders, and buyers [3].

However, there have been no similar investigations for hip genetic quality in the other breeds recorded in the voluntary OFA database. In addition, there are no investigations of the long-term genetic changes in OFA elbow scores, as there have been for hip scores in the USA. Several studies have characterized the genetic underpinnings of both HD and ED in the British [4], Swedish [8], Finnish [9] and Dutch [10] dog populations. A multiple-trait model, which incorporates both genetic and environmental correlations, would provide more accurate estimates of genetic parameters like heritability and EBVs than a single trait model [4], [11]. In this study, in order to extend EBV-related techniques to a wide range of breeds, and combining hip and elbow scores together, we used a bi-variate mixed model to estimate the genetic parameters for both hip and elbow scores, so that we could provide more accurate selection criteria against ED and HD for dog owners and breeders.

## Materials and Methods

### Dogs

By the end of 2010, there were 1,066,596 hip and elbow conformation records from 329 breeds reported in the voluntary (public) OFA database, including the following attributes for each dog: name, registration id, breed, sex, birth date, sire id, dam id, trait name, trait score, and test date (www.offa.org). After removing the replicate dogs, 1,065,218 records remained. For accuracy sake, the breeds with less than 1000 hip and elbow records were removed, as well as crossbreds. Chondrodysplastic breeds including Havanese, Cardigan Welsh corgi, Miniature Australian Shepherd, Pembroke Welsh Corgi, Cavalier King charles Spaniel, Bichon Frise, Cocker Spaniel, Soft Coated Wheaten Terrier and English Cocker Spaniel were also excluded from the analysis because it is unclear that the genetic basis for hip and elbow malformation in these breeds is identical to that for nonchondrodysplastic breeds. The dogs scored before 24-month or after 60-month of age were removed from the analysis [3]. Only the dogs born after 1970 were considered, and the records scored before 1974 and after 2009 were eliminated. As most of the dogs were scored at 2 years of age, the dogs born after 2007 were also removed to satisfy the score date before the end of 2009. For the dogs with multiple scores for one trait, their last records were retained.

For elbow score, additional edits were implemented. Only normal dogs or dogs with secondary osteoarthritis/degenerative joint disease were considered. The breeds with no elbow score variation, or less than ten ED dogs, were removed from the analysis, which led to 21 breeds remaining. As well, the individuals with birth year prior to 1988 were grouped into 1988.

After data editing and the imposition of quality control conditions, 895,864 records from 772,035 dogs in 74 breeds remained, including 760,455 hip scores and 135,409 elbow scores. Among these were 123,829 dogs with a record for both traits.

Pedigree records on 1,491,113 dogs were built from the attributes specified as registration identification (ID), sire ID and dam ID, which were extracted from the public OFA database. After removing dogs that were replicates, or with breeds eliminated, or with mis-recorded sex (such as female sire or male dam), birth date (recorded as born earlier than a parent), or recorded as both father and mother, there were 1,264,422 individuals in the pedigree, of which 492,387 were ancestors without phenotypic records. The trait records and pedigrees used in this retrospective study were available in the public domain and no live animals were used. We did not access the private records deposited in the OFA registry.

### Hip and Elbow Scores

According to the OFA, dogs were scored into seven categories for hip conformation: excellent, good, and fair hip conformation, borderline, then mild, moderate and severe hip dysplasia and each score was assigned a numerical value from 1 (excellent hip conformation) to 7 (severe hip dysplasia). The first three categories (excellent, good and fair) are often considered as “normal” dogs. The last three categories (mild, moderate and severe) are considered as “dysplastic” dogs. The score strategy has changed in four different time periods, which was explained in our previous study in detail [3]. For elbow conformation, dogs were categorized into four groups as “Normal”, “Degenerative Joint Disease I, II and III”, with the corresponding numerical scores from 1 to 4. Thus for both hip and elbow conformations, lower scores are better and lower EBVs are more desirable. Due to the unbalanced distribution of elbow scores, log, square root and reciprocal transformations of the scores were also performed.

### Statistical Model

A bi-variate linear mixed animal model (BLM) was employed to estimate the genetic parameters for hip and elbow scores. The model can be illustrated in matrix notation accordingly:
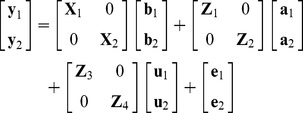
where **y_1_**, **y_2_** were the vectors of observations for hip and elbow scores, respectively, for elbow scores, **y_2_** also represented log, square root or reciprocal transformations besides the original observations, and were noted as BLM (LOG), BLM (SQRT) and BLM (INV) besides BLM (O), respectively; **b_1_** and **b_2_** were vectors of fixed effects for the two traits, where **b_1_** included breed, sex, test year period, test year nested within test year period, birth year, and 4 age groups [3]; the effects of **b_2_** were the same as **b_1_** except for test year period; **a_1_** and **a_2_** were the vectors of additive genetic effects, *i.e.* breeding values; **u_1_** and **u_2_** were the vectors of combination effects of test year and test month, referred as random effects; and **e_1_** and **e_2_** were the vectors of random residual effects. **X**_1_, **X**_2_, **Z**_1_, **Z**_2_ and **Z**_3_, **Z**_4_ were incidence matrices associating **b_1_**, **b_2_, a_1_, a_2_** and **u_1_**, **u_2_**, respectively. It was assumed that 
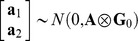
, 
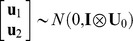
, and 
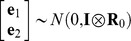
, where **A** is the matrix of additive genetic relationships, constructed as a block diagonal matrix partitioned by breed in our case, since only purebred dogs were included in the analysis, **I** is an identity matrix, **G_0_**, **U_0_** and **R_0_** were the variance-covariance matrix of additive genetic effects, combination effects of test year and month, and residuals between **y_1_** and **y_2_** respectively, 
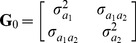
, where 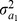 and 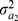 were the additive genetic variances for the two traits, and 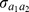 was the covariance between additive genetic effects of the two traits, 
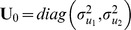
, 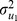 and 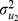 were the variances of combination effects of test year and month for the two traits, 
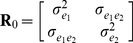
, where 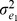 and 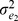 were the residual variances for the two traits, and 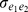 was the covariance between residuals of the two traits. Average information restricted maximum likelihood method (DMU Package [12], [13]) was used.

### Accuracy of Breeding Value

The accuracy of the EBVs was derived from their prediction error variance (PEV) and the additive genetic variance (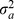) using the following formula: 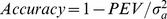.

### Inbreeding Coefficient

The individual inbreeding coefficient equals the corresponding diagonal element of the **A** matrix minus one. The earliest ancestors of the dogs in our study were born in 1970. There were no further pedigrees that could be traced back for dogs born between 1970 and 1973, so these animals were therefore considered as founders with inbreeding coefficient of zero.

### Ethics Statement

The study was conducted on the public data available from http://www.offa.org. There is no requirement for formal institutional approval for statistical and genetic analysis.

## Results

### Basic Statistics

**Figure 1:**
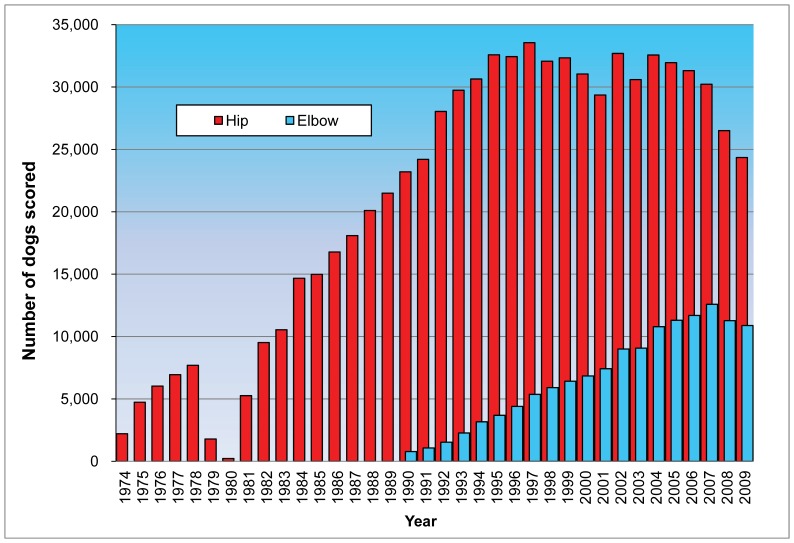
Number of dogs with hip and elbow scores during 1974 to 2009. Red bar indicates the number for hip scores, and blue bar for elbow scores. There were 760,455 and 135,409 hip and elbow scores, respectively, in 74 dog breeds collected by the Orthopedic Foundation for Animals (OFA) during this period.

**Figure 2:**
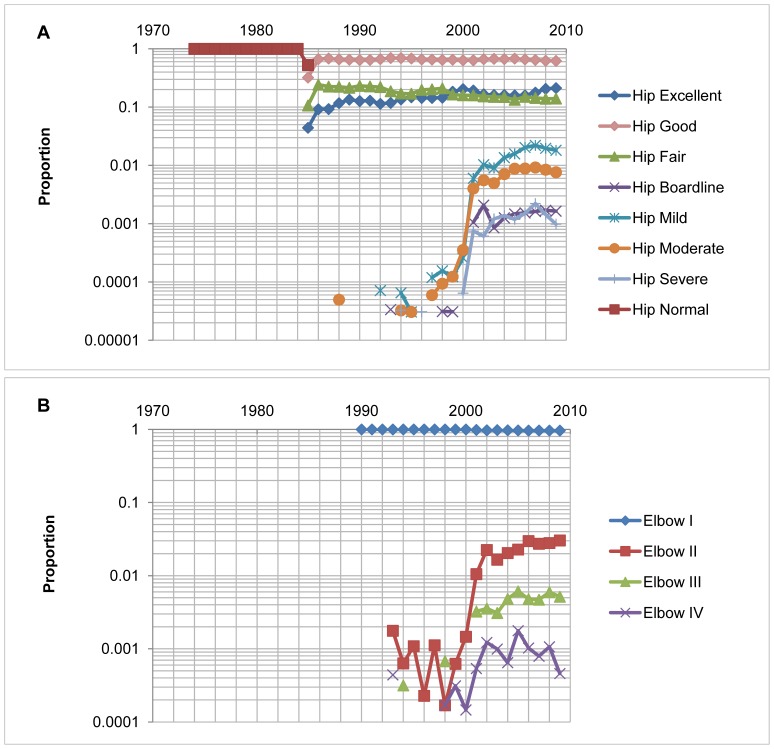
Distribution of hip and elbow scores released between 1974 and 2009. Figure 2A: Only three categories of hip scores (Excellent, Good and Fair) were jointly released as Normal between 1974 and 1985 and separately reported after 1985. There was no release on other categories before 2000. Then, all categories were released and reported separately; Figure 2B: Elbow I meant “Normal”, Elbow II, III, IV meant osteoarthritis (degenerative joint disease) level I, II and III respectively. Few elbow scores were released except for category “Normal” before 2000. The reports were heavily biased against reporting poor hip and elbow scores in the first 30 years.

Since 1974, the number of dogs scored on hip and elbow conformation by the OFA continually increased each year. The number of scores reached its peak in 1997 for hip joint conformation, and in 2007 for elbow joint conformation (Figure 1). The detailed distributions of hip and elbow joint conformation scores for each breed are displayed in Table S1. The average scores for hip and elbow joint conformation were 2.05 and 1.03, respectively (Table 1). Following dichotomization, the average incidences of HD and ED were 0.83% and 2.08%, respectively (Figure 2). The incidences of HD in the 74 breeds ranged from 0.07% to 6%, with Boykin Spaniel and St. Bernard having the highest incidences, and Siberian Husky and Afghan Hound, the lowest. The incidences of ED, specified as degenerative joint disease, ranged from 0.5%–8% in the 21 breeds, with the Rottweiler having the highest incidence and Rhodesian Ridgeback, the lowest.

**Table 1:** The means and standard deviations (SD) of hip and elbow scores. These statistics and number of scores (N) are characterized by sex and category of age in months.

		Hip joint score			Elbow joint score		
		N	Mean	SD	N	Mean	SD
**Sex**	Female	481,434	2.05	0.63	87,214	1.02	0.17
	Male	279,021	2.05	0.63	48,195	1.03	0.21
**Age**	24	185,751	2.04	0.62	41,454	1.03	0.19
	25–29	271,459	2.05	0.62	50,458	1.02	0.18
	30–36	149,287	2.07	0.63	22,784	1.03	0.19
	37–60	153,958	2.07	0.65	20,713	1.03	0.21
**Total**		760,455	2.05	0.63	135,409	1.03	0.19

### Breed, Age and Sex Effect

**Figure 3:**
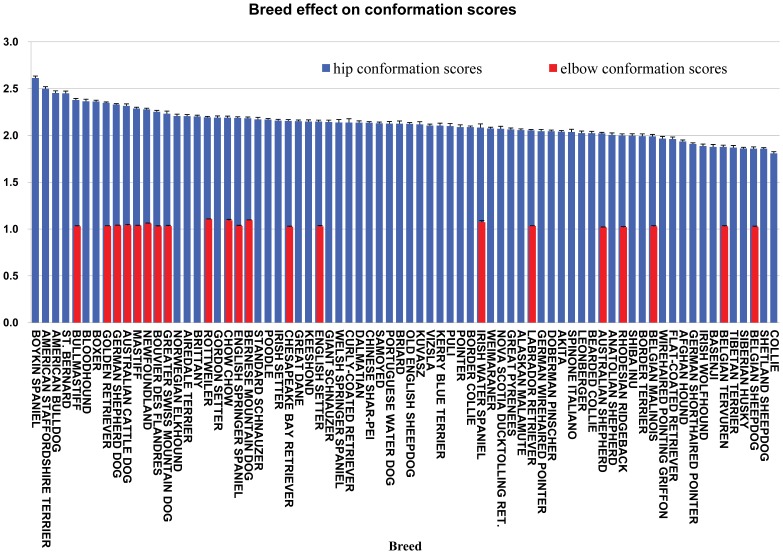
Breed effects on hip and elbow scores and their standard errors. A breed was required to have a minimum of 10 dogs recorded as “degenerative joint disease I or II, or III” to be included in the analysis, which led to only 21 breeds remaining for elbow scores evaluation. All the 74 breeds satisfied the requirement on hip scores.

After correction of the other influential factors, such as sex, age group and birth year, the breed effect significantly influenced the hip and elbow scores in our dataset (P<0.01) (Figure 3). The hip scores measured on 37–60 month-old-dogs were significantly higher (P<0.01) than those measured on 24 or 25–29 month-old-dogs, which was consistent with our previous publication dealing with HD in the US Labrador Retriever population [3]. However, the age group effect was not significant for the elbow score. For hip score, the sex effect, precluding the influences of the other factors, was not significant (P>0.05), which agreed with the results of our previous study on Labrador Retriever hips [3]. However, the elbow scores for males were significantly higher than those for females; the difference between male and female effects was 0.0082±0.0010 (P<0.01).

### Genetic Parameters and Genetic Trends

The estimated genetic parameters for hip and elbow conformation scores by using the BLM are illustrated in Table 2. As shown, for hip scores, the estimates of genetic and residual variance were 0.0849 and 0.2880, respectively. Consequently, the heritability was estimated as 0.23±0.0025. In contrast, for elbow scores, the genetic variances for the original elbow scores, their log, square root and reciprocal transformations were 0.0056, 0.0021, 0.0008 and 0.0009, respectively. The estimated heritabilities were highly similar (0.16±0.0055). The genetic correlation between hip and elbow conformation scores was 0.12±0.018, and the environmental correlation was 0.08±0.0043. The correlations between EBVs derived from different transformations for elbow score approximated to 1,which indicated that the differences were undetectable between data transformations for elbow score compared to the original scores.

**Table 2:** The estimated genetic parameters and their standard deviations (SD) under different models*.

Parameters	BLM(O)	BLM(LOG)	BLM(SQRT)	BLM(INV)
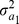	0.08488±0.00096	0.08487±0.00096	0.08487±0.00096	0.08487±0.00096
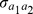	0.00270±0.00039	0.00166±0.00024	0.00104±0.00015	−0.0011±0.00016
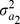	0.00559±0.00019	0.00209±0.00007	0.00083±0.00002	0.00094±0.00003
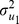	0.00057±0.00005	0.00057±0.00005	0.00057±0.00005	0.00057±0.00005
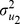	0.00002±0.00000	0.00000±0.00000	0.00000±0.00000	0.00000±0.00000
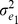	0.28800±0.00089	0.288±0.00089	0.28800±0.00089	0.28800±0.00089
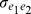	0.00749±0.00039	0.00471±0.00024	0.00293±0.00015	−0.00320±0.00016
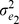	0.02908±0.00019	0.01063±0.00007	0.00425±0.00002	0.00479±0.00003
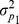	0.3728±0.00063	0.37280±0.00063	0.37280±0.00063	0.37280±0.00063
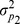	0.03467±0.00013	0.01272±0.00005	0.00509±0.00002	0.00574±0.00002
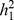	0.22760±0.00245	0.22760±0.00245	0.22760±0.00245	0.22760±0.00245
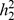	0.16130±0.00548	0.1644±0.00546	0.1633±0.00547	0.16470±0.00545
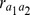	0.12400±0.01806	0.12530±0.01788	0.12480±0.01794	−0.12550±0.01785
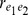	0.08187±0.00433	0.08520±0.00433	0.08393±0.00433	−0.08620±0.00433

* All the models are performed with original hip score and different transformations of the elbow score. The transformations include Log, square root (SQRT) and inverse (INV). In each transformation of elbow score, the hip score remains the status of original score. The model of Original (O) was performed on the original scores on both hip and elbow. BLM (O) means the bi-variate linear model with original hip and elbow conformation scores as observations; BLM (LOG) means the bi-variate linear model with original hip conformation scores and log transformations for elbow scores as observations; BLM (SQRT) means the bi-variate linear model with original hip scores and square root transformations for elbow scores as observations; BLM (INV) means the bi-variate linear model with original hip scores and inverse transformations for elbow scores as observations. 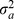 is the additive genetic variance, 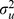 is the variance of combination effects of test year and test month, 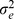 is the residual variance, 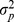 is the phenotypic variance, 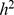 is the heritability, subscript 1 means the trait of hip joint conformation scores, subscript 2 means the trait of elbow joint conformation scores. 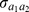 is the additive genetic covariance between these two traits, 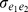 is the residual covariance between them, 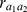 is the additive correlation between them, 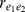 is the residual correlation between them.

**Figure 4:**
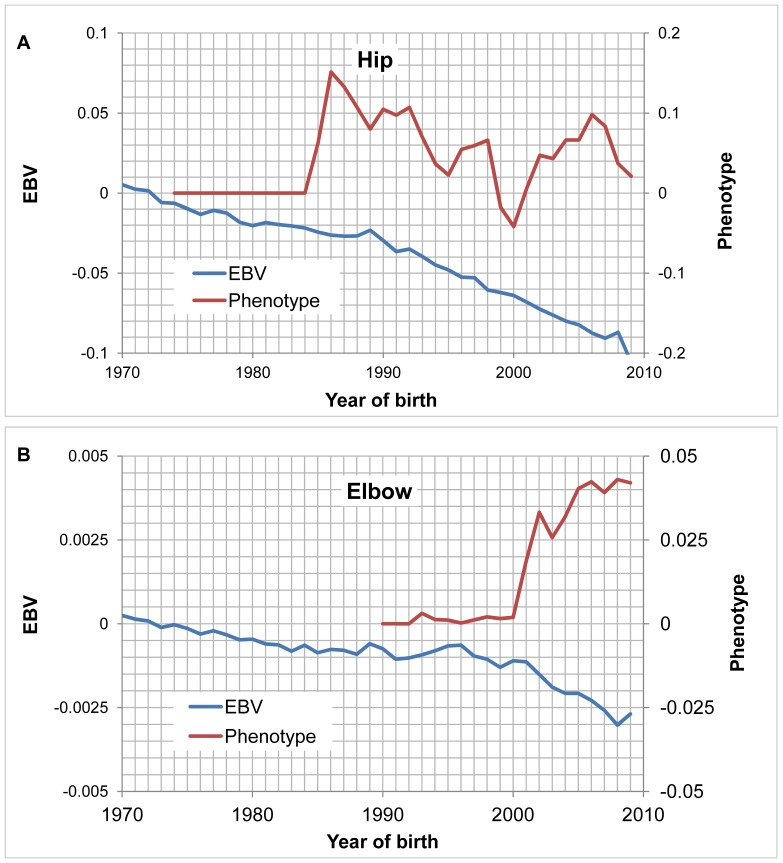
The phenotypic and genetic trends of hip and elbow scores. Figure 4A shows the trends for hip scores while figure 4B shows the elbow scores. The trend lines represent the mean within each year for phenotype (the vertical axis on the right) and estimated breeding value (EBV; the vertical axis on the left) for the 1,264,422 dogs born between 1970 and 2009. The phenotype was presented as the deviation from the score (1) of the best hip or elbow.

Although phenotypes of hip and elbow scores fluctuated over time, the EBVs exhibited steady improvements (Figure 4) between 1970 and 2009 for hip scores, reflecting consecutive improvement year over year in HD genetic quality during this period. This was accompanied by only very modest improvements in ED genetic quality between 1996 and 2009. These represented a total increment of 0.1 and 0.0021 units of EBV for hip and elbow scores, which were equivalent to 16.4% and 1.1% of the total phenotypic standard deviations of each trait, respectively. By approximately fitting an normal distribution, we can make interpretation on the change in incidence of HD. The mean and the variance of the distribution were 2.05 and 0.63. The observed incidence of 0.83% corresponds to a score of 3.56 from the normal distribution. After the mean is reduced by 0.1 hip score, the same cutoff corresponds to incidence of 0.52%. The elbow score does not have a normal distribution. The interpretation on the incidence change is not trivial.

The genetic improvement exhibited variation among breeds (Table S2). For instance, for HD, some breeds presented steady genetic improvement like Akita, German Shorthaired Pointer, Labrador Retriever, Siberian Husky, and Weimaraner. Some breeds showed no improvement, like Basenji, Bloodhound, Curly-Coated Retriever, and Boxer, while the St. Bernard showed deteriorating hip genetic quality. In contrast, for ED, breeds like Labrador Retriever and Golden Retriever presented obviously steady, but modest, improvements in elbow EBVs after about 1993. However, the Rottweiler showed explicit deterioration in elbow genetic quality.

**Figure 5:**
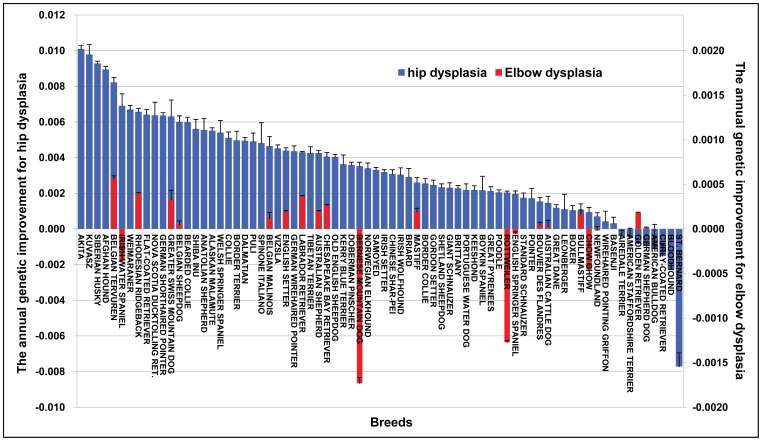
The annual genetic improvement in each breed over 40 years. The annual genetic improvement is represented by the average increments of estimated breeding value (EBV) per year and its standard errors. The blue bar indicates the genetic change for hip dysplasia on the vertical axis on the left, the red bar indicates the genetic change for elbow dysplasia on the vertical axis on the right. A breed was required to have a minimum of 10 dogs recorded as “degenerative joint disease I or II, or III” to be included in the analysis, which led to only 21 breeds remaining for elbow scores evaluation. All the 74 breeds satisfied the requirement on hip scores.

The regression coefficient of EBVs plotted against birth year for each breed profiled the annual genetic improvement (AGI) (Figure 5). The AGIs of HD and ED for Labrador retriever, for example, were 0.0043 and 0.00037, respectively. The breed with the most rapid AGI against HD was Akita, having an AGI of 0.010, followed by Kuvasz (0.0098), Siberian Husky (0.0093), Afghan Hound (0.0089) and Belgian Tervuren (0.0082). Of note, St. Bernard had the highest annual genetic deterioration rate for HD with an AGI of −0.0077, while Rottweiler had the highest annual genetic deterioration rate for ED of −0.0013, which was even higher after 2000. Considering the formula for selection response *R = iσ_p_h* ^2^/*L*, where the selection response (*R*), heritability (*h* ^2^), the phenotypic deviation (*σ_p_*), and the generation interval (*L*) for Labrador Retriever were 0.0043, 0.23, 0.6, and 4.3 approximately [3], [5], then the selection intensity (*i*) against HD, i.e. the decline, was estimated at 13% over 40 years. Similarly, if taking *R*, *h* ^2^, *σ_p_*, and *L* for elbow score as 0.00037, 0.16, 0.19, and 4.3, the selection intensity against ED was calculated as 5% only over the same time period. In addition, for the same phenotypic score, the EBVs presented substantial variations, for example, the EBVs ranged from −0.98 to 0.47 within the category of “Excellent” for hip conformation, and from −0.12 to 0.61 within the category of “Normal” for elbow conformation, which indicated a potential advantage of selection based on EBV over phenotype scores.

**Figure 6:**
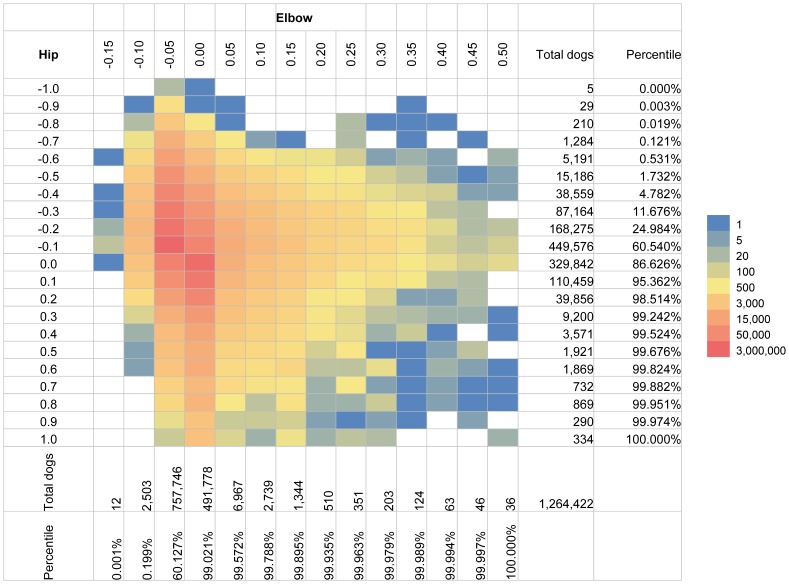
The joint and marginal distribution of breeding values of hip and elbow scores. Estimated breeding value (EBV) of hip scores is displayed on the vertical axis and EBV of elbow scores is plotted on the horizontal axis for 1,264,422 dogs born between 1970 and 2009. The marginal distributions of hip and elbow EBVs are indicated by both the total number of dogs and percentile in each category.

An examination of the joint distribution of EBV for both hip and elbow scores (Figure 6), revealed low correlation (0.21) between these two traits. This finding was consistent with the low genetic correlation of 0.12 calculated from variance components. A closer examination of genetic correlations between these two traits for each breed based on their EBVs showed a range of 0.06–0.52 (Figure S3), with Chesapeake Bay Retriever the highest, and Belgian Malinois the lowest.

### Accuracy of EBV and Selection

The joint distributions of EBVs and their corresponding accuracies were illustrated in Figure S1 and S2 for hip and elbow scores, respectively. The correlation coefficients between EBV and their accuracies for hip and elbow scores across all breeds were −0.24 and −0.03 respectively, which indicated that better (lower) EBVs tended to have higher accuracies. The correlation coefficients between hip score EBVs and accuracies within breeds varied from −0.46 (Weimaraner) to 0.12 (Curly-Coated Retriever) (data not shown), of which, only two breeds (Curly-Coated retriever and St. Bernard) showed positive correlations. This suggests that for most American dog breeds, there has been selection against HD. In comparison, the correlation coefficients between elbow score EBVs and their accuracies were not as strong as those for hip score, having a range of −0.29 (Chesapeake bay retriever) to 0.19 (Chow chow). This indicates that across breeds, the selection intensity against ED has been low.

### Genetic Pattern in Labrador Retriever

Since Labrador Retriever was the only breed involved in both our previous and current studies, we calculated the correlation of hip score EBVs between these two studies for the common dogs. The high correlation coefficient (0.94 and 0.93 for Pearson correlation and Spearman rank correlation, respectively) implied the high concordances between them. The average accuracy in previous study was 0.24±0.16. In the current study undertaken 2 years later, the accuracy increased by 8.3% to an average of 0.26±0.16.

Analysis of the updated EBV showed that improvement in accuracy was a reflection of those dogs with more recorded progeny in recent years. We evaluated the EBVs and accuracies for common parental dogs (299 male dogs and 356 female dogs) whose 437 progeny were born after 2007 (In our previous study, we did not include the dogs born after 2007, so there should be increases in the accuracy of EBVs for these common parents as a consequence of the acquisition of more progenies’ hip and elbow performances). For the male dogs, the correlation of EBVs between these two studies was only 0.82, yet the accuracy increased from 0.45±0.19 to 0.53±0.19. For the female dogs, the correlation was 0.92, and the accuracy increased from 0.37±0.10 to 0.42±0.08.

In addition, we compared the prediction ability for a single individual’s hip and elbow scores by using its parents’ phenotype and EBVs, respectively. The prediction ability is reflected in the concordance between an individual’s performance and its prediction from parents. The correlations between an individual’s hip and elbow score and its phenotypic prediction from its parental scores (equivalent to half of the sum of the parents’ scores) for Labrador Retriever, were 0.12 and 0.02, respectively. However, the correlations between an individual dog’s EBV and its EBV prediction from its parents were both as high as 0.91. It can be concluded that EBVs present powerful prediction ability for an individual’s performance. Therefore, selection against HD and ED based on EBVs would confer a much needed advantage over selection based on phenotype alone, as is currently most often the case.

### Inbreeding

**Figure 7:**
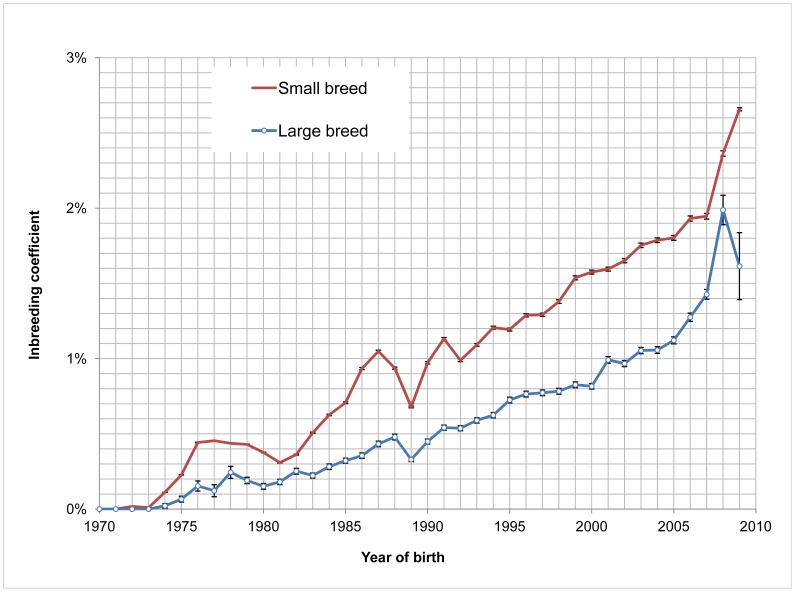
Trends of inbreeding coefficients over 40 years for small- and large-population breeds. The small and large population breeds were separated by a threshold value of 50,000 dogs. Breeds with 50,000 or less dogs were defined as small population breeds, while breeds with more than 50,000 dogs were the large population breeds, which included the Labrador Retriever, Golden Retriever, German Shepherd Dog, and Rottweiler.

We found the level of inbreeding in breeds with a small population was higher than the one in large population breeds (Figure 7). The investigated breeds were classified into two categories: the breeds with fewer individuals (with 50,000 or less dogs) and those breeds with more individuals (with more than 50,000 dogs, containing Labrador Retriever, Golden Retriever, German Shepherd Dog, and Rottweiler) born between 1970 and 2009. Breeds with smaller populations had an average inbreeding coefficient of 0.0082±3.8*10^−5^, which is about twice of the inbreeding level in large population breeds (0.0044±2.5*10^−5^). The average increments of inbreeding coefficients were 0.0665% and 0.045% per year over the past forty years for breeds with smaller and larger populations, respectively.

## Discussion

### Bias in the Data Base

The phenotypic data reported in the public domain of the OFA registry is biased for HD and ED by the voluntary nature of the reporting. Fewer radiographs would be submitted for the individuals from undesirable families and for the individuals that have been pre-screened as badly affected to reduce the cost of the official certification, and some breeders presumably do not wish the hip and elbow scores of their dogs to be revealed. Inspection of the breed summary statistics for HD and ED in the OFA registry show that the incidence of dysplasia among the breeds studied here is often far higher than revealed in the individual animal data to which we had access. Nevertheless, considering the individual’s pedigree information and removing effects of environmental factors in the evaluation by employing EBVs would partially decrease the bias of using the radiographic phenotype only in breeding decisions [3], [14], [15]. Clearly, the more complete the pedigree and phenotype information included in the estimation of the breeding values, the more accurate the breeding value estimates will be.

### Genetic and Environmental Characteristics of the Two Joint Disorders

A comprehensive analysis across all the breeds was implemented, rather than a separate analysis within each breed [4], [16], which can compensate for the deficiency in voluntarily reporting bias from OFA by considering pedigree information and removing all effects of environmental factors such as breed, age, sex et al. As well, our goal was to describe the average genetic quality of hip and elbow conformation of the US pure breed dog populations over the period studied, so that we could draw general conclusions regarding the effect of voluntary orthopedic registries. To describe the individual genetic parameters, like heritability, for every breed was beyond the scope of our study. However, we do describe the change in genetic quality for breeds that do not follow the average improvement in genetic quality.

The estimated heritability of hip scores in this study fell in the range of reported estimates between 0.1 and 0.68 as reviewed by Zhu et al [1]. Of note, besides the OFA registry, there are three other evaluation schemes for hip conformation. The Fédération Cynologique Internationale (FCI) scheme is widely used in most mainland European countries, Russia, South America, and Asia [17], [18], the British Veterinary Association/Kennel Club (BVA/KC) scheme is used in Britain, Ireland, Australia and New Zealand [18], [19], and the Verein für Deutsche Schäferhunde (SV) scheme is used in Germany (http://www.offa.org) [18]. Based on information released by BVA/KC, the heritability of hip score on a log scale was estimated as 0.35 in UK Labrador retrievers [5], [20], and ranged from 0.28 to 0.48 in various UK dog breeds [16]. Estimates of heritability reported in papers based on the BVA/UK registry are based on the entire population of dogs’ hip phenotypes rather than only publicly accessible records, while our analysis is based only on publicly accessible records. As well, based on the BVA scheme, a range of 0.14–0.25 was also reported by using a linear model in a cohort of Australian German shepherd dogs [21]. It was indicated that the discrepancy in estimates of heritability for hip scores was attributed to distinct evaluation schemes and populations. There are no published estimates of heritability for any trait recorded in the OFA registry that is based on the entire data set. Hip phenotypes based on the extended-hip ventrodorsal radiographic projection, as used by these schemes, do not disclose as much hip laxity as the PennHip™ [22] radiographic projection or the dorsolateral subluxation test [23]–[26]. When estimates of heritability have been made based on these latter tests, the estimates have been higher. The heritability of elbow score estimated in our study was consistent with the spectrum of 0.06–0.31 reported in the literatures across different breeds [4], [9], [10], [16], [20].

The average genetic correlation between hip and elbow scores was estimated as 0.12 in this study, which was lower than the estimates of 0.40–0.42 in UK Labrador retrievers [4], [20], and between the estimates of 0.23 and 0.07 in the Swedish Rottweiler and Bernese Mountain Dog, respectively [8]. The low genetic correlation suggested that it’s not always efficient to aim for genetic improvement for HD by indirectly selecting against ED, and vice versa. The residual correlation between hip and elbow scores was 0.08, which estimated the effect of similar risk factors for ED and HD, such as nutrition and exercise [27], [28]. Fortunately, the influence was not great. The disparity between genetic correlations for HD and ED for each breed suggested that a specific selection strategy should be considered for each breed. For a breed with high genetic correlation between hip and elbow scores like the Chesapeake Bay Retriever, selection on either trait could benefit the other (especially for elbow scores) because selection against HD could compensate for the scarcity of records on elbow scores. However, selection based on both traits jointly would result in more rapid and balanced genetic improvement, than selection based on one trait alone.

A significant difference in the breed effect on the hip and elbow scores has been detected in our dataset. The reported disparity of breed effect might reflect the breed-specific nature of susceptibility to HD and ED. However, the other causative factor, severe reporting bias in public OFA individual records that has been discussed above, can’t be ruled out. No significant difference in susceptibility to HD between male and female was revealed in this population of US dogs. However, males were more frequently affected by ED than females, possibly because of their faster growth rate and greater over-all size.

On average, we found a limited genetic improvement on hip scores from 1970 to 2009 in the 74 breeds, and on elbow scores from 1996 to 2009 in the 21 breeds we studied, indicating a general trend for alleviation of hip and elbow dysplasia. The genetic improvement of HD reached up to 16.4% of the phenotypic standard deviation, which was in accordance with our previous results in Labrador Retrievers [3]. Of note, the improvement of ED was trivial, contributing to only 1.1% of the phenotypic standard deviation. We ascribed this to the relatively late collection of elbow scores (the collection of elbow scores was initiated from 1990), the total number of dogs scored (was around only one sixth of that of hip scores), and the reliance on osteoarthritis to reveal any elbow abnormality other than ununited anconeal process on the flexed lateral radiographs projection. Radiographs are insensitive to early osteoarthritic related changes in proximal limb joints. Selection based on elbow score has been available for only a short time. This implied the importance of selection against ED and the necessity of collection of many more elbow conformation scores, followed by its regular genetic evaluation.

It is worthy to note that in our study, the genetic improvements for HD and ED were underestimated to some degree due to the reporting bias from OFA. There was no osteoarthritic record (with a high value) reported in the first 15 years, and the number of osteoarthritic records continually increased in last ten years. Also, as the phenotypes were collected nation wide, there were many sources of variation, e.g. breed, age, sex and other environment factors. The reported phenotype did not represent a stable trend over last 40 years. However, by using a mixed model, which took all environmental factors into account and linked the dogs reported recently and the one reported 40 years ago together by tracing the pedigree, the EBVs still illustrated a clear fact that the OFA approach improved the genetic quality, on average over all breeds, for HD and ED. Further, the genuine breeding values of the older dogs should be worse than estimated, as their own osteoarthritic records were not voluntarily reported, which would lead to underestimated genetic improvements.

A wide range of variation in the magnitude and rate of genetic change exhibited within each breed, indicated distinct breeding selection goals or lack thereof, selection intensity was carried out according to individual breed characteristics and requirements, or that health committees of some breed clubs are encouraging and educating breeders and owners concerning the heritability of HD and ED. The trend of steady, if irregular, improvement for HD was illustrated in breeds like the Akita, German Shorthaired Pointer, Labrador Retriever, Siberian Husky, and Weimaraner, which highlighted the long-term selection strategies against HD in US dogs for these breeds. Of note, analysis of the St. Bernard hip records indicated a genetic deterioration. It is also possible that St. Bernard owners are more inclined to reveal the hip scores of their dogs to the public domain than owners of other breeds. The genetic improvement for ED was demonstrated in only some breeds including Labrador Retriever and Golden Retriever after about 1993. The genetic deterioration of Rottweiler elbow scores was observed after about 1993. After profiling the trends of genetic improvement including the current status within each breed, the extent of improvement, the generally poor improvement speed, and comprehensively considering the knowledge of breed characteristics and breeding requirements, it would be extremely helpful to establish a breeding goal for each breed. For instance, based on the status of genetic deterioration, a strengthened selection against HD for St. Bernard could be planned in future. And if we would like to speed up the general genetic improvement in the Labrador Retriever, we could increase the current improvement rate (13%) by dropping a larger proportion of dogs with bad hips status from the breeding pool because it is a breed with a large population.

A reasonable selection based on EBVs for HD and ED could improve hip and elbow conformation in these pure breed dogs. The selection signal demonstrated by the joint distribution of EBV and accuracy for HD and ED supports this contention. The dogs with better phenotypes i.e. lower EBVs for HD and ED were reported and were predisposed to be bred more frequently, which led to more progeny and therefore higher accuracies of the derived EBVs. Other considerations are that EBV had more substantial variation than the phenotype and the correlation of EBV between parents and their progeny was significantly higher than that based on their phenotypes. This demonstrated inefficient selection based on phenotype, especially for elbow score, which is exactly consistent with our finding of trivial genetic improvement against ED.

In our present study, we only considered the direct genetic heritability, which is the biggest and most important variance component for hip scores. Maternal and litter effects are reportedly not negligible when predicting the EBVs of dogs for hip scores [5]. Therefore, in a future study, besides the direct genetic effect, maternal genetic and litter effects could be considered in the bi-variate model.

Compared to our previous results [3], a slight increase was detected for the accuracy of EBV of hip scores in this study for Labrador Retriever, which was mainly attributed to the introduction of a bi-variate model (the effect of data expansion can be ignored since the size (437) of the data increase was small), which was also consistent with the fact that the elbow scores did not provide enough information for prediction of EBV of hip scores in light of the relatively low genetic correlation between them. However, for the breed with high genetic correlation between hip and elbow scores, the utilization of bi-variate model would confer greater accuracy of prediction of EBV, and therefore accuracy of selection against both HD and ED [4], [11].

### Selection Strategy

The data suggested that selection based on the combination of the phenotypic score and relatives’ information such as that of parents and siblings has led to limited but steady improvement in HD and ED scores over the past 40 years [3]. Since both HD and ED were moderately heritable quantitative traits, selection based on EBVs could increase accuracy of selection against HD and ED, thereby hastening genetic improvement, over the relative inefficiency of selection based on dogs’ phenotypic scores and visual inspection of their pedigrees. The same conclusion was also reached in other studies [4], [5], [16], [20]. The possibility of elevating inbreeding raises concern as selection pressure increases. Although the average inbreeding coefficients in the dog population were not very high yet, they still constantly increased. Avoiding mating between close relatives (parent and offspring and full and half siblings at least) has to be recommended always. Inbreeding coefficients can be married with the HD/ED EBVs in selecting dogs for breeding. With more elbow score data collected in the future, a selection index to jointly reduce both HD and ED should be developed for each breed, to achieve better efficiency and balanced improvement. It seems that the OFA might provide individual EBVs for every dog in the registry without revealing their individual hip and elbow scores. This would require extensive education of the dog owning community through on line information, seminars, phone consultation, and veterinary continuing education.

With the availability of sequencing technology and high-density whole genome single nucleotide polymorphism (SNP) canine mapping arrays, the associated markers and critical genes underpinning HD and ED will be identified [29], [30]. The molecular genetic information could be combined into a selection scheme to increase the accuracy and lower the incidence of disease more rapidly, and likely more effectively, than that based on EBVs alone [31]–[34]. Recently, a promising method called genomic selection has been developed based on the genomic EBV and successfully applied to livestock animal breeding [35]. Genomic selection can increase the accuracy of EBVs, and decrease the generation interval by selecting individuals at the early stage of life to accelerate the genetic improvement, because the genomic information can be used to predict susceptibility to HD [36]. We predict that genomic selection having practical application in selection against HD and ED will be available in the future.

### URLs

The breeding values, accuracies and inbreeding coefficients of the 1,264,422 dogs of 74 American breeds will be released at the World Wide Web as follows:

www.vet.cornell.edu/research/bvhip.

### Ethics Statement

No live animals were used in this study.

## Supporting Information

Figure S1 **The joint and marginal distribution of estimated breeding values and accuracies for hip scores.** Estimated breeding values (EBV) of hip scores were displayed on the vertical axis and accuracies were plotted on the horizontal axis for the 1 M dogs born between 1970 and 2007. The marginal distributions of EBVs and accuracies were indicated by both the total number of dogs and percentile at each category. The dogs on the upper right with lower EBV (better hip) and higher accuracy were the most ideal for breeding against hip dysplasia.
(TIFF)

Figure S2 **The joint and marginal distribution of estimated breeding values and accuracies for elbow scores.** Estimated breeding values (EBV) of elbow scores were displayed on the vertical axis and accuracies were plotted on the horizontal axis for the 1 M dogs born between 1970 and 2007. The marginal distributions of EBVs and accuracies were indicated by both the total number of dogs and percentile at each category. The dogs on the upper right with lower EBV (better elbow) and higher accuracy were the most ideal for breeding against elbow dysplasia.
(TIFF)

Figure S3 **The correlations between EBV of hip and elbow joint conformation scores for 21 dog breeds.** The number of non-normal records (degenerative joint disease I, II, and III) for each breed was required to be larger than 10, which led to only 21 breeds remaining.
(TIFF)

Table S1 **The detailed distributions of hip and elbow joint conformation scores for 74 American dog breeds.**
(XLSX)

Table S2 **The yearly genetic improvement over past 40 years for hip and elbow dysplasia for 74 American breeds.**
(XLSX)
