# Correction: Monitoring Hip and Elbow Dysplasia Achieved Modest Genetic Improvement of 74 Dog Breeds over 40 Years in USA

**DOI:** 10.1371/annotation/92e1aa00-169b-45dc-9866-61034e061f6d

**Published:** 2013-10-16

**Authors:** Yali Hou, Yachun Wang, Xuemei Lu, Xu Zhang, Qian Zhao, Rory J. Todhunter, Zhiwu Zhang

One of the funding sources for this article was omitted. The funding source can be found below.

The Program for Changjiang Scholar and Innovation Research Team in University (IRT1191) 

