# Supplementary figures and images for: Monitoring Hip and Elbow Dysplasia Achieved Modest Genetic Improvement of 74 Dog Breeds over 40 Years in USA
Source: PLoS One. 2013 Oct 4;8(10):e76390. doi: 10.1371/journal.pone.0076390 (PMC3790730; doi:10.1371/journal.pone.0076390)

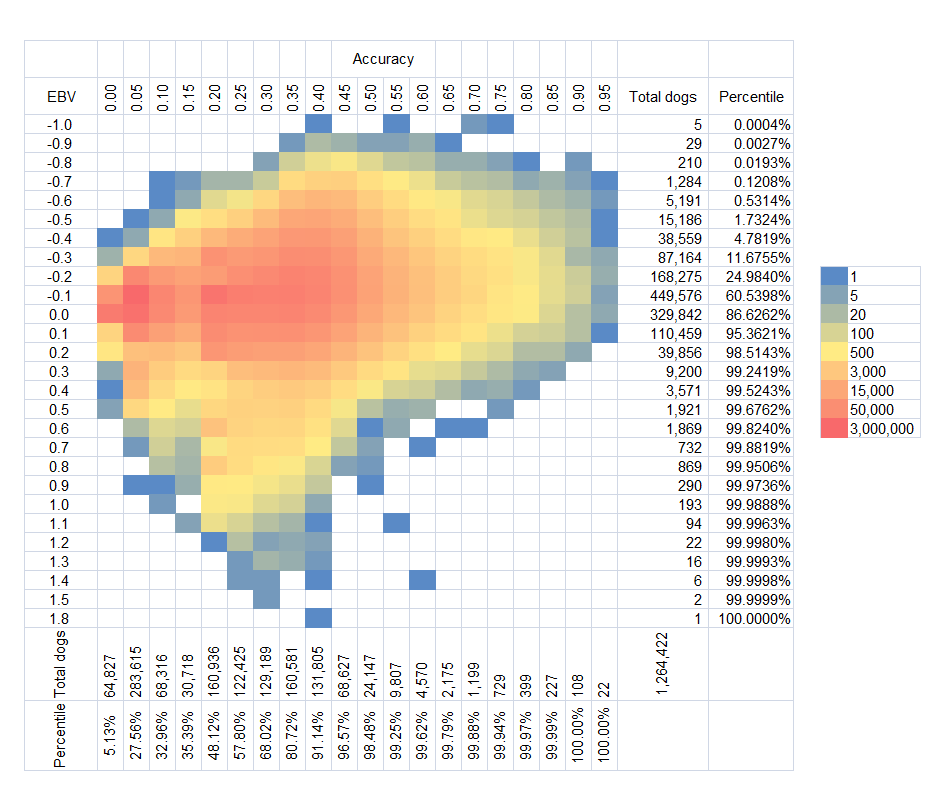

Supplement: Figure S1 — The joint and marginal distribution of estimated breeding values and accuracies for hip scores. Estimated breeding values (EBV) of hip scores were displayed on the vertical axis and accuracies were plotted on the horizontal axis for the 1 M dogs born between 1970 and 2007. The marginal distributions of EBVs and accuracies were indicated by both the total number of dogs and percentile at each category. The dogs on the upper right with lower EBV (better hip) and higher accuracy were the most ideal for breeding against hip dysplasia. (TIFF) [file pone.0076390.s001.tiff]

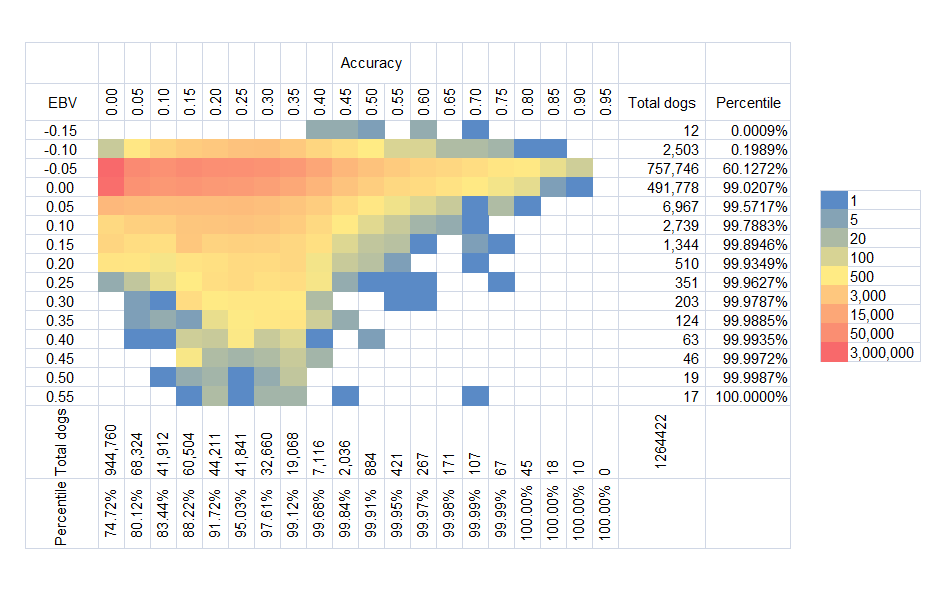

Supplement: Figure S2 — The joint and marginal distribution of estimated breeding values and accuracies for elbow scores. Estimated breeding values (EBV) of elbow scores were displayed on the vertical axis and accuracies were plotted on the horizontal axis for the 1 M dogs born between 1970 and 2007. The marginal distributions of EBVs and accuracies were indicated by both the total number of dogs and percentile at each category. The dogs on the upper right with lower EBV (better elbow) and higher accuracy were the most ideal for breeding against elbow dysplasia. (TIFF) [file pone.0076390.s002.tiff]
